# Dr Yvonne Edmonstone: Formerly Consultant Psychiatrist and Psychotherapist, New Craigs Hospital, Inverness

**DOI:** 10.1192/pb.bp.116.054494

**Published:** 2016-12

**Authors:** Jane Morris, Tim Agnew, Heather Ireland

**Figure:**
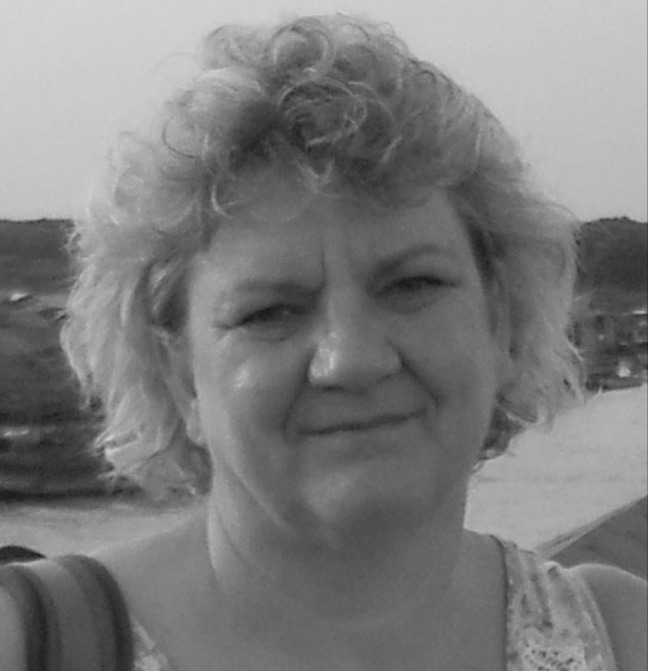


Dr Yvonne Edmonstone – who died of breast cancer on 17 September 2015 at the age of 52 – was the clinician responsible for the creation of the National Health Service (NHS) Highland Eating Disorder Service and one of the founder members of the North of Scotland Managed Clinical Network for Eating Disorders. In 2009 she helped pave the way for the opening of the Eden Unit, Scotland's first NHS in-patient unit for adults with eating disorders. As well as her enormous contribution to the field on a local and regional level, she was a key player on a national scale, as part of the core group which set up Eating Disorders Education and Training Scotland, and as a long-standing contributor to the Scottish Eating Disorders Interest Group. On her retirement, grateful patients produced a tribute video and – in appreciation of her contribution to the field – she received an award from BEAT, the UK charity supporting people with eating disorders.

Although the area of eating disorders was where she focused most of her professional energy, Yvonne had a broad interest in the application of psychotherapy to mental disorder. She was a valued supervisor for the South of Scotland Cognitive Behavioural Therapy Course and an early adopter of the interpersonal psychotherapy (IPT) model, her case reports being preserved as exemplars by IPT course accreditors. Yvonne was also a keen advocate of dialectical behaviour therapy and a key member of the Highland team working in that field. She was an expert psychotherapist whose advice was sought by other clinicians because of her impressive clinical acumen and compassionate, common-sense approach. As well as her clinical commitments, she contributed to more academically focused endeavours. She co-authored a chapter on psychotherapy in the award-winning manual Research Methods in Psychiatry^1^ and was an author of the Scottish Intercollegiate Guidelines Network guideline on the non-pharmaceutical management of depression in adults.

Born on 20 March 1963, Yvonne, as a schoolgirl, was torn between her artistic talents and passion for becoming a doctor. In adult life her creativity expressed itself not only through her psychotherapeutic practice but also in her talent for organising (and participating in!) social events. She enthusiastically planned and enjoyed holidays, lunches and trips to music festivals with family and friends until only weeks before her death.

Yvonne undertook dual training in general adult psychiatry and psychotherapy. She qualified in 1985 with an MBChB from the University of Aberdeen. Her special interest in eating disorders involved both clinical and research work in Edinburgh's Cullen Centre. On completion of her training, she moved back to her beloved Highlands, where she poured her heart and soul into building the Highland Eating Disorder Service. She became a Fellow of the Royal College of Psychiatrists in 2014.

Humane and thoughtful, Yvonne was held in the highest regard and affection by colleagues and patients alike. Her easy, yet infectiously enthusiastic manner and sense of humour were valued not only by those who knew her professionally, but also by everyone who came into contact with her socially.

Yvonne demonstrated extraordinary generosity of spirit, both professionally and personally. She was someone who really would go the extra mile. She encouraged, mentored and otherwise inspired many clinicians from different professional backgrounds to fulfil their potential. She always had time for her colleagues despite her frenetic schedule, while her engaging nature brightened many a dull day at work and many a social occasion besides.

Yvonne's impact on local, regional and national psychotherapy and eating disorders services cannot be underestimated. However, her greatest legacy is her beloved family – the four children she raised with her husband Alistair and the close relationship she enjoyed with her parents. Her ability to maintain such a truly productive work–life balance is an example for us all. She is sadly missed.
